# Cholecysto-duodenal and cholecysto-colic fistulae identified intraoperatively during laparoscopic cholecystectomy in a surgical hub

**DOI:** 10.1308/rcsann.2025.0054

**Published:** 2025-07-15

**Authors:** C R Smith, A Valnarov-Boulter, R Kerwat

**Affiliations:** Lewisham and Greenwich NHS Trust, UK

**Keywords:** Laparoscopic cholecystectomy, Ambulatory surgical procedures, Elective surgical procedures, Gallstones, Fistula

## Abstract

Laparoscopic cholecystectomy is the standard treatment for symptomatic gallstone disease and is often performed in elective settings. To address surgical backlogs, the United Kingdom has adopted off-site elective surgical hubs designed for high-volume, low-complexity procedures. We describe a case of a young woman undergoing laparoscopic cholecystectomy at a surgical hub, in whom two cholecysto-enteric fistulae – cholecysto-duodenal and cholecysto-colic – were unexpectedly identified intraoperatively. Both were successfully managed laparoscopically. This rare intraoperative finding, in a low-risk, preoperatively uncomplicated case highlights the importance of surgical vigilance and reinforces the need for surgical hubs to be equipped with appropriate expertise, instruments, and escalation pathways to manage unanticipated intraoperative complexity.

## Background

Gallstone disease is one of the most common conditions requiring surgery, with more than 60,000 cholecystectomies performed annually in the United Kingdom (UK).^1^ Although laparoscopic cholecystectomy is typically considered a routine elective procedure, cholecysto-enteric fistulae (abnormal communications between the gallbladder and the gastrointestinal tract) are encountered in 0.5%–0.74% of cases.^2,3^ These rare fistulae usually arise from chronic or recurrent gallstone-related cholecystitis.^4^ Repeated episodes of inflammation can lead to adhesions with adjacent viscera (such as duodenum or colon) and progressive erosion of the gallbladder wall, ultimately resulting in fistula formation.^4^

Surgical management typically involves cholecystectomy with fistula division and repair, increasing the procedural complexity. Preoperative diagnosis is achieved in only 8%–17% of cases, with most fistulae first identified intraoperatively, requiring unanticipated adjustments to surgical approach.^5,6^

To address elective surgery backlogs, the UK has established elective surgical hubs (off-site centres designed for high-volume, low-complexity procedures).^7^ Although cholecystectomy is well suited to this setting, appropriate case selection and intraoperative preparedness are crucial. Unexpected intraoperative findings, such as cholecysto-enteric fistulae, may arise even in patients assessed as low risk.

This case report describes the successful laparoscopic management of dual cholecysto-enteric fistulae in an elective surgical hub, underscoring the importance of surgical vigilance and robust contingency planning within elective surgical hubs.

## Case history

A woman in her early 30s with a history of obesity (body mass index 40kg/m^2^) and a known left-sided renal calculus was scheduled for elective laparoscopic cholecystectomy at an off-site elective surgical hub. The procedure took place approximately 7 months after an initial emergency department attendance with acute gallstone-related cholecystitis.

One year prior to her acute presentation, a routine abdominal ultrasound had identified a solitary 18mm gallstone, with no other abnormalities. At that time, she was asymptomatic.

She first presented acutely with severe epigastric pain, fever (38.4°C), nausea and mild exertional dyspnoea. Clinical examination revealed tenderness in the right upper quadrant and epigastrium without jaundice. Blood tests showed a mildly elevated C-reactive protein (CRP) level (60mg/L), with normal full blood count, liver function tests and serum lipase. A computed tomography (CT) scan of the abdomen and pelvis showed a distended gallbladder with peri-cholecystic fat stranding, in keeping with uncomplicated acute cholecystitis. She was discharged with safety netting and a course of oral co-amoxiclav.

Three days later, she re-presented with worsening abdominal pain and ongoing fever. Examination revealed right upper quadrant tenderness and a positive Murphy’s sign. Inflammatory markers were significantly raised (CRP 280mg/L) and repeat CT imaging again showed findings consistent with acute cholecystitis with no reported features of gallbladder perforation, biliary dilatation, fistula or other complications. She was admitted for intravenous antibiotic therapy and underwent an ultrasound scan, which demonstrated a thickened gallbladder wall (6.7mm) and a large, impacted calculus in the gallbladder neck. Following clinical and biochemical improvement, she was discharged after 5 days and placed on the elective waiting list for laparoscopic cholecystectomy.

Over the following months, she underwent routine nurse-led preoperative assessment. Blood tests confirmed resolution of inflammation. As per standard local protocol for patients living with obesity, she completed a 2-week liver shrinkage diet in preparation for surgery. She remained clinically well and asymptomatic at the time of surgery, which proceeded as an elective laparoscopic cholecystectomy at the off-site surgical hub.

Following safe peritoneal entry and laparoscopic port placement, intraoperative findings were consistent with chronic calculus cholecystitis. The gallbladder appeared thick-walled and oedematous, with a large, impacted calculus located in the neck. Two distinct cholecysto-enteric fistulae were identified.

**Figure 1:**
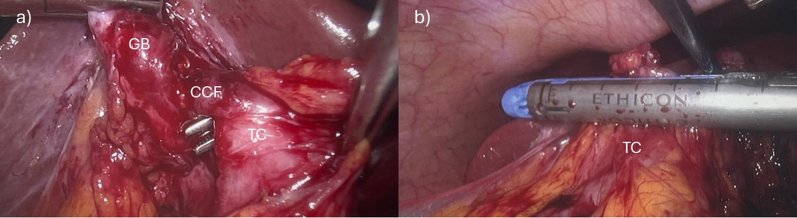
Intraoperative photographs of the cholecysto-colic fistula. (a) Fistulous communication between the body of the gallbladder and the transverse colon. (b) Following division of the fistula; the colonic side has been closed using a laparoscopic stapler (Echelon Flex 60, blue cartridge). CCF = cholecysto-colic fistula, GB = gallbladder, TC = transverse colon

First, the transverse colon near the hepatic flexure was found to be densely adherent to the gallbladder. A fistulous connection between the between the body of the gallbladder and the colon was suspected. Dense adhesions were carefully released using laparoscopic scissors. A cholecysto-colic fistula was confirmed and divided, with division on the gallbladder side, away from the colon. The colonic side was closed using a laparoscopic stapler (Echelon Flex™ 60, blue cartridge; Ethicon, Johnson & Johnson) (Figure 1).

**Figure 2:**
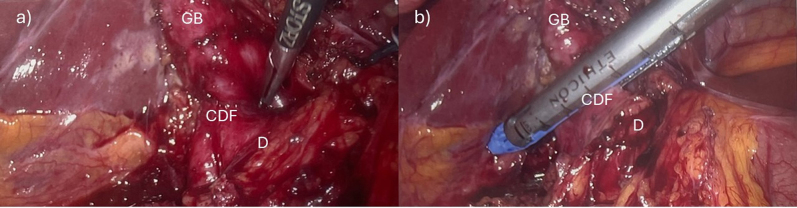
Intraoperative photographs of the cholecysto-duodenal fistula. (a) Fistulous connection between the body of the gallbladder and the proximal duodenum. (b) Division of the cholecysto-duodenal fistula using a laparoscopic stapler (Echelon Flex 60, blue cartridge). CDF = cholecysto-duodenal fistula, GB = gallbladder, D = duodenum

Further dissection revealed a second fistula, this time between the body of the gallbladder and the proximal duodenum. The cholecysto-duodenal fistula was divided using the same laparoscopic stapling device, with division maintained on the gallbladder side (Figure 2).

Cholecystectomy was then performed. The critical view of safety was obtained, and the cystic duct and artery were clipped and divided. The gallbladder was removed, and a surgical drain was placed in the gallbladder fossa before closure.

The patient recovered well in the immediate postoperative period and was discharged on the day of surgery with oral analgesia, a 7-day course of co-amoxiclav, and a 2-week course of omeprazole (40mg daily). She was scheduled for expedited follow-up in the surgical ambulatory unit on postoperative day 3.

At this review, she was clinically well with minimal haemoserous drain output. Blood tests revealed a raised CRP level (218mg/L) but were otherwise unremarkable. Given the elevated inflammatory marker and complicated nature of the surgery, a CT scan of the abdomen and pelvis was performed, which demonstrated expected postoperative inflammatory changes in the gallbladder fossa, with no evidence of intra-abdominal collection, leak or obstruction. The drain was removed, and a second follow-up was arranged for postoperative day 14.

At this review, inflammatory markers had returned to normal, and the patient remained clinically well. On specific questioning, she reported two additional episodes of abdominal pain between her emergency admission and the operation, which had been managed in primary care but were not detected during the preoperative assessment or recorded in the hospital records by the time of surgery. A final routine outpatient review was scheduled and is currently awaited.

Histopathological examination of the resected gallbladder confirmed features consistent with chronic cholecystitis with cholelithiasis.

## Discussion

**Figure 3:**
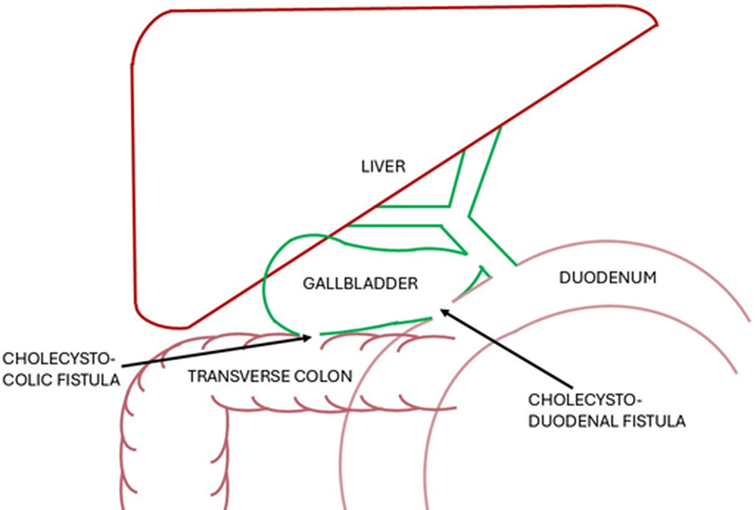
Illustration of relevant anatomy, demonstrating the coexistence of both cholecysto-duodenal and cholecysto-colic fistulae in the same patient. Created by CR Smith.

To our knowledge, ours is the first published case of a cholecysto-enteric fistula successfully managed in an elective surgical hub. Cholecysto-enteric fistulae are a rare complication of gallstone disease. The most common type is the cholecysto-duodenal fistula accounting for approximately 75%–80% of cases, followed by cholecysto-colic fistulae, reported in 8%–26.5%.^5,^^8^ The coexistence of both types of fistulae in a single patient is exceptionally rare. Figure 3 illustrates the anatomical findings of both fistulae identified intraoperatively in this case.

In a large retrospective review by Chowbey *et al* involving 12,428 patients undergoing laparoscopic cholecystectomy, only two cases of multiple fistulae were reported – one with two cholecysto-duodenal fistulae, and another with both cholecysto-duodenal and cholecysto-colic fistulae.^2^ Similarly, in a separate cohort of 10,588 patients from China, no cases of multiple cholecysto-enteric fistulae were reported, underlining the rarity of our case.^5^

A brief review of recent published cases reinforces this. One case described an emergency laparotomy for gallstone ileus, in which both fistulae were discovered and treated alongside enterotomy and stone extraction.^9^ Another case, more comparable with ours, involved the unanticipated intraoperative discovery of both fistulae during elective laparoscopic cholecystectomy. However, in that report, after fistula division, repair was performed using laparoscopic suturing, in contrast to our use of stapling devices.^10^

Most reported cases involve elderly patients with a long-standing history of symptomatic gallstone disease.^5^ By contrast, our patient was young and had a relatively short disease course, making this an unexpected intraoperative finding in an otherwise low-risk case. This underscores the importance of maintaining a high index of suspicion for complex pathology even in patients who appear suitable for day-case off-site laparoscopic cholecystectomy.

The growing use of elective surgical hubs in the UK for high-volume, low-complexity operations raises important considerations for patient safety and surgical planning. This case illustrates the limitations of current preoperative assessment pathways. Although the patient had two further primary care presentations for abdominal pain between referral and surgery, these were not visible to the surgical team owing to the lack of integrated records. In the context of gallstone disease, tailored pre-assessment, such as direct questioning about symptom recurrence could improve risk stratification and case selection.

Intraoperative preparedness is equally critical. The availability of laparoscopic stapling devices from emergency stock enabled safe laparoscopic management of both fistulae, avoiding conversion to open surgery. This highlights the need for regular review of contingency equipment and for ensuring that senior surgical expertise is available on-site to manage unexpected complexity, particularly as surgical hubs expand the procedures they offer.

Postoperative care must also be robust. In this case, the use of a surgical ambulatory unit allowed timely follow-up, including early blood tests and CT imaging, facilitating safe discharge and early identification of potential complications. Surgical hubs should be supported by clear postoperative pathways that allow urgent review and investigation when required.

## Conclusions

This case contributes to the limited literature on multiple cholecysto-enteric fistulae, highlighting the rare co-occurrence of both cholecysto-duodenal and cholecysto-colic fistulae in a single patient. Despite modern imaging, such findings may remain undetected preoperatively, especially in patients assessed as low risk. This reinforces the need for continued surgical vigilance, even during seemingly routine elective procedures.

As elective hubs become integral to surgical recovery strategies within the National Health Service, this case offers a timely reminder that efficiency must be balanced with adaptability, safety and appropriate escalation systems. With the right safeguards in place, even rare and complex intraoperative findings can be managed successfully in off-site elective settings.
